# Larval Wound Therapy: Possibilities and Potential Limitations—A Literature Review

**DOI:** 10.3390/jcm12216862

**Published:** 2023-10-30

**Authors:** Dariusz Bazaliński, Joanna Przybek-Mita, Kamila Pytlak, Daria Kardyś, Adrian Bazaliński, Marek Kucharzewski, Paweł Więch

**Affiliations:** 1 Podkarpackie Specialist Oncology Centre, Specialist Hospital in Brzozów, 36-200 Brzozów, Poland; dbazalinski@ur.edu.pl (D.B.); kamila.pytlak@interia.pl (K.P.); 2 Institute of Health Sciences, College of Medical Sciences, University of Rzeszów, 35-959 Rzeszów, Poland; joprzybek@ur.edu.pl; 3 Postgraduate Nursing and Midwifery Education Centre, 35-083 Rzeszów, Poland; 4 Frederic Chopin Provincial Clinical Hospital No. 1, 35-055 Rzeszów, Poland; daria.kardys10@gmail.com; 5 Student Scientific Association of Nurses, Sanok State University, 38-500 Sanok, Poland; adi.baz.2002@wp.pl; 6 Collegium Medicum, Jan Długosz Częstochowa University, 42-200 Częstochowa, Poland; kucharzewskimarek@poczta.onet.pl; 7 Institute of Health Protection, State University of Applied Sciences in Przemyśl, 37-700 Przemyśl, Poland

**Keywords:** patient-centered care, *Lucilla sericata* larvae, MDT, health problems, chronic wound, nursing care

## Abstract

Patient-centered care (PCC) is recognized as a standard in healthcare for determining high quality. Honoring patients’ values, experiences, needs, and preferences in devising, coordinating, and delivering care underscores the enhancement of the therapeutic rapport between patients and healthcare providers. Wound treatment involves a multi-stage process encompassing diagnostics and local wound dressing, which reduces the risk of infection through a coordinated interdisciplinary team. Within this team, nurses undertake specific professional functions and roles. The implementation of local therapy using innovative and scientifically substantiated methods may be hindered by a deficit of knowledge or inappropriate knowledge among staff and patients themselves. This study presents the challenges concerning the care of patients with chronic wounds treated using Lucilia sericata larvae, based on a review of the current scientific literature. A critical analysis of the literature spanning from 2002 to 2022 was conducted using the Medline, PubMed, Cochrane, and Termedia databases, employing keywords such as “maggot debridement therapy” in relation to acceptance and perception. As a result of the preliminary selection, 472 papers were identified, of which 12 publications were included in the development of this concept. The acquired data were organized and presented in the concluding section in the form of tables, accompanied by descriptions and references to individual studies. Negative psychological and somatic sensations were among the most prominent challenges among patients treated with Maggot Debridement Therapy (MDT). Pain related to peripheral ischemia or infection in this group of patients requires pain prophylaxis, including hyperalgesia and allodynia, in order to improve method tolerance. On the other hand, augmenting patients’ understanding of MDT diminishes negative emotions, reinforces positive behaviors, and mitigates anxiety levels. MDT constitutes an effective and safe method. Its widespread use for chronic wounds requires substantial knowledge among healthcare professionals and patient education, along with that of their caregivers, to develop a positive attitude.

## 1. Introduction

Despite the development of medicine and access to advanced treatment methods, the problem of difficult-to-heal and chronic wounds continues to be an interdisciplinary challenge for healthcare workers. Unfavorable prognoses regarding the epidemiology of lifestyle diseases such as diabetes, cardiovascular diseases, obesity, and an aging society predispose to an increase in the number of patients with hard-to-heal wounds. Their treatment involves a multi-stage process, starting with broadly understood diagnostics based on biochemical, microbiological, imaging, and occasional histopathological tests conducted by a prepared interdisciplinary team, where the nurse performs specific functions and roles [1,2,3]. Topical wound management concepts (TIMERS and the wound hygiene consensus) emphasize wound debridement, which should be performed as quickly as possible to reduce the bacterial load and encourage physiological granulation tissue formation [4,5]. Both Polish [6] and global guidelines [7] recommend the MDT (Maggot Debridement Therapy) method for the treatment of hard-to-heal wounds of various etiologies, which was approved by the American Food and Drug Administration (FDA) in 2004 [8]. In recent years, an increase in interest in this method of treatment has been observed in Poland, in which many doctors and nurses see greater opportunities for a more expeditious, safer, and cost-effective way of achieving the debridement and revitalization of hard-to-heal wounds. It is recognized that the pioneer of maggot therapy, as it is applied today, is R.A. Sherman, who, in 1990, opened a sterile laboratory at the Veteran Administration Hospital Medical Centre, in Long Beach, California. His team carried out a prospective study involving patients with pressure wounds following spinal cord injury, where it was shown that, in comparison to conservative methods, wound debridement was more effective and required less time, while safety measures and sterile larva culture were maintained [9,10]. The use of biodebridement in the world in the era of “antibiotic resistance” is gaining more and more recognition among experts dealing with the problem of chronic wounds. The issue of biodebridement is often raised by authors from Germany, England, the United States, Turkey, and Nigeria. In the last decade, the medical larvae of *Lucilia sericata* have been referred to as miraculous “medical maggots” due to their various biochemical properties that stimulate repair processes in the wound [11]. In Poland, in 2023, an expert team of the Polish Wound Management Society (PTLR) developed the first national recommendations regarding the use of medical larvae, and the method was formally named Larval Wound Therapy (LWT) [12].

Therapy using *Lucilia sericata* larvae is a well-tested and widely used method in the local treatment of wounds with confirmed clinical effectiveness [7,13]. Wound debridement is contingent upon the presence of larvae in the wound, which liquefy and eliminate devitalized tissues. The stimulation of repair processes is linked to the excretions and secretions produced by maggots. The use of larvae in the debridement and stimulation of repair processes in wounds should be associated with the empirical knowledge and skills of healthcare professionals providing care and treatment. Although the concept of maggot treatment seems to be easy. In practice, it requires commitment from the caregivers, including adherence to a protocol of actions taken in the wound and for the patient [14]. Multi-Drug Resistant Organisms (MDRO) have become a serious threat to civilization, stimulating the search for more effective methods of destroying microorganisms. Insightful observations and research by Sherman and Pechter on the elimination of bacterial flora, including MRSA (methicillin-resistant Staphylococcus aureus), by larvae placed in the wound, opened up new opportunities for researchers and clinicians all over the world [8,9]. An analysis of wounds before and after the application of medical maggots proves the promotion of wound healing on many levels. Hence, the interesting element of MDT is a broad chemical action based on the secretion and excretion of specific enzymes and their correlation with antibacterial activity (Lucilin, Lucifensin, Lucifensin II, and MAMP (Alpha-methoxyphenol), seraticin antibiofilm (Chymotrypsin), anti-inflammatory (excretions/secretions—ES), synergism with selected antibiotics, and immunomodulatory functions [10,15,16]. Malone et al. determined the presence of biofilm in 78.2% of chronic wounds, which significantly affects healing processes [16]. The antimicrobial effect of maggots has been confirmed in the case of bacteria highly resistant to antibiotics and the ability to produce biofilm. The elimination of this form of pathogens is very beneficial due to their high resistance to the penetration and activity of the human immune system and antibiotics. The use of larvae significantly minimizes the bacterial load, especially *Pseudomonas aeruginosa* and *Staphylococcus aureus*, both of which are often resistant to antibiotics [17,18]. It is estimated that, in Europe, approximately 15,000 patients per year are qualified for local wound treatment procedures using MDT [19,20]. In Poland, MDT therapy is not reimbursed by the National Health Fund (NFZ), however, taking into account the possibility of conducting therapy at home and significantly shortening the time of the local treatment process, it is deemed safe and cost-effective in overall assessments [21,22].

Personal factors on the part of the patient and systemic barriers may, to some extent, inhibit the implementation and dissemination of MDT in the process of the local treatment of wounds of various etiologies. Patient-centered care (PCC) is recognized as a standard in healthcare that establishes high quality. Honoring the values, experiences, needs, and preferences of the patient in planning, coordinating, and providing care determines the improvement of the therapeutic relationship between the patient and healthcare providers. In the model approach, practices are used to build positive experiences for patients in the field of care provided. On the basis of these practices, dimensions were defined (respecting patient preferences, coordination and integration of care, involvement of family and significant others, information, education, ensuring comfort, emotional support, continuity of care after discharge, and access to care) [22,23,24]. Implementing thoughtful measures in professional practice can reduce fears of implementing biodebridement and minimize the potential health issues associated with MDT.

The aim of the study is to review the literature dealing with the subject of the perceptions and potential health problems related to MDT in a group of patients with hard-to-heal wounds.

## 2. Materials and Methods

**Figure 1:**
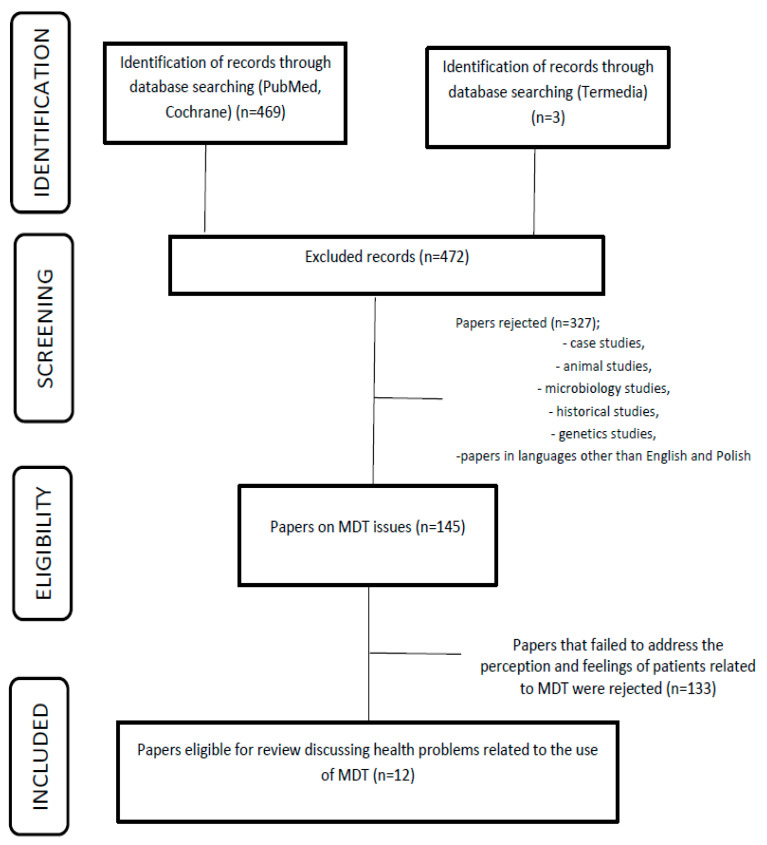
Protocol of inclusion of works for analysis.

The study employed a qualitative literature review covering the years 2002–2022, using the Medline, PubMed, Cochrane, and Termedia databases. The keywords “maggot debridement therapy”, “acceptance”, “perception”, and “pain” were applied in the literature search to identify the available literature. The databases were analyzed, and 472 duplicates were identified (Figure 1) through manual reference searches of the selected manuscripts. Subsequently, 327 sources that were not directly pertinent to the intended analysis, such as case studies, case series, genetic, microbiological, and animal studies, were excluded. In the subsequent phase, 145 works were selected, encompassing descriptions of potential issues encountered during MDT therapy. Among these, 133 studies were eliminated, as they did not address the specific subjects of interest, namely, acceptance, perception, and health problems. Twelve papers were included in the final analysis, including original articles and randomized trials (see Table 1). The data collection process consisted of two stages. In the first stage, the titles and abstracts of manuscripts in both English and Polish were screened to assess their relevance. Eligible articles were then advanced to the second stage of selection, during which, the full texts were scrutinized to determine their inclusion. This process was carried out by two members of the research team and finally approved by the project leader.

For reporting, the study adopted the PRISMA protocol and utilized a flowchart scheme to enhance the visual presentation of the literature review process. This protocol allowed for a quality analysis of the selected literature. All pertinent data from the included studies were entered into a table.

### 2.1. Patient-Centered Care (PCC) Model

The patient-centered care (PCC) model is recognized as the standard approach in healthcare to improve its quality. The essence of PCC is to honor the patient’s values, experiences, needs, and preferences in planning, coordinating, and providing care at every stage [23]. The patient and healthcare providers’ therapeutic relationship is central to this issue. The implementation of the model into practice improves care outcomes, shortens hospitalization time, lowers costs, and increases patient satisfaction with care [24]. In terms of the model, there are certain practices that foster a positive patient experience regarding the care provided. Based on these practices, eight dimensions were established (honoring patient preferences, coordination and integration of care, involvement of family and significant others, information and education, ensuring comfort, emotional support, continuity of care after discharge, and access to care) [36].

### 2.2. Lucilia sericata Larvae and Topical Protocols for the Treatment of Difficult-to-Heal and Chronic Wounds

A wound that is not subject to physiological repair processes is defined as difficult to heal. Damage or destruction of the skin and/or subcutaneous tissue that takes more than 6–8 weeks to heal or an area that does not decrease by 20–40% after 2–4 weeks of treatment is defined as a chronic wound [6]. Damage to the common integument tissue represents only a fraction of the patient’s health problems and can significantly impact their quality of life, potentially leading to feelings of hopelessness, shame, and fear of infection and sepsis. Accompanying pain, effusion, malodor, and the fear of limb loss often serve as strong motivating factors for seeking alternative local treatment methods, provided symptoms of depression and resignation have not occurred [15,16]. Wound debridement, specifically tissue debridement, constitutes the foremost and most vital element in the TIMERS concept. It reduces the risk of infection by eliminating necrotic tissue, thus creating conditions conducive to granulation and epithelialization. The choice of wound debridement methods and the timing and location of their application are influenced by various factors, including the wound’s location, the depth of the tissue damage, the amount of exudate, and the patient’s overall condition [6]. While there are no clear and direct guidelines for cleaning a wound when repair processes are inhibited, the method for eliminating devitalized tissue is multifactorial. It is contingent on various factors, including the wound’s location, depth of damage to tissue structures, amount of exudate, coexisting pain, and the patient’s preferences [4,5]. Mechanical wound debridement (such as rubbing, scraping, plucking, or cutting) represents the simplest, most cost-effective, and fastest method of biofilm removal when administered by trained medical personnel [5]. However, in most chronic wounds with concomitant biofilm, more advanced interventions are necessitated, which may include “sharp” or surgical debridement, biological autolytic processes, and potentially the implementation of controlled negative pressure (NPWT) in local wound therapy [8]. The removal of necrotic tissue from the wound enhances oxygen availability to healthy tissues, facilitates the migration of fibroblasts and keratinocytes, and physically eliminates pathological microorganisms, thus reducing the likelihood of their further proliferation [10]. Medical maggot therapy combines elements of both mechanical and autolytic methods. Indications for MDT are expanding every year. This expansion is observed not only in the treatment of diabetic foot ulcers (DFU), pressure injuries (PI), and chronic vascular wounds of the lower limbs, but also in complex infected postoperative wounds.

*Lucilia sericata* (Meigen) larvae (Diptera: Calliphoridae) are the most commonly used maggots and are sterilized before application to ensure that no further bacterial infections are introduced during treatment [37,38]. These biological materials can be applied directly to the wound, typically at an average rate of 5–10 larvae per cm^2^, or in a biobag. It is preferable to apply medical maggots for 3–4 days [39]. In a systematic review of clinical studies from 2000–2014, Sun et al. analyzed 12 comparative studies, including 6 randomized controlled trials. Based on the analysis of these 12 studies, the authors concluded that larval therapy was more effective and efficient in debriding chronic ulcers compared to conventional treatments, such as hydrogel and active dressings [40]. While the use of MDT in clinical practice may not become standard for an extended period due to the limited quality of scientific evidence and various implementation challenges [37], every effort should be made to engage healthcare professionals involved in the care and treatment of patients with chronic wounds, encouraging them to consider this method in their practice.

### 2.3. Health Problems in the Care of a Patient Qualified for MDT

Living with a chronic wound can have a substantial impact on an individual’s physical, psychological, social, and spiritual well-being. Financial costs can also burden individuals living with wounds and their families. The success of the therapeutic and care activities performed is guaranteed by following the planned nursing process, developed on the basis of interviews, physical examinations, patient observations, analyses of medical records, and questionnaire assessments. The aim of nursing care is to improve the condition or maintain it at the current level when the patient’s treatment options have been exhausted. The nurse’s participation in the process of local wound treatment is the main element of the therapeutic function. In Poland, nurses acquire competence to treat chronic wounds as a part of pre- and post-graduate education. Knowledge of the recommendations and guidelines of scientific societies (EWMA, European Wound Management Association, Polish Society of Wound Treatment, PTLR) increases the possibility of implementing new therapeutic methods into practice [6,12,14].

Among patients and some medical staff, there is still a belief about low effectiveness, the experimental nature of the method, or negative visual and pain sensations that cause fear, reluctance, and lack of acceptance [8,21,37]. Stereotypically, larvae are still associated with ugliness, vermin decomposing carrion, and unconventional treatment. The limited and often inaccurate societal messaging concerning the possibilities, simplicity, and cost-effectiveness of the method might predispose negative attitudes among healthcare representatives and potential patients themselves. Perception includes visual and olfactory sensations and images, as well as beliefs and attitudes about therapy. Potential reasons why patients avoid MDT may be related to disgust [41], repulsive mental images [42], pain [16,39], and specific unpleasant odors [40]. Patients’ experiences include their psychosomatic feelings during and after therapy. The gathered data are presented in Table 2 and Table 3, accompanied by descriptions that are grounded in evidence-based medicine (EBM).

### 2.4. Cost of Larval Therapy Application

In Poland, larval therapy is becoming more and more popular among representatives of medical professions, although some still believe that it is an unconventional method. The lack of reimbursement from the payer (National Health Fund) does not significantly affect the possibilities of its use due to the relatively low prices of the larval product. It is assumed that the PTLR recommendations developed in May 2023 will strengthen and contribute to a broader perception of the method among clinicians, especially those conducting professional activity in outpatient care [12]. In the list of services offered, only a dozen or so centers in the country offer the treatment of wounds with larvae, hence, the availability of larval therapy is still unsatisfactory. In Germany [11] and England, MDT is a reimbursed service, and the production of dressings with larvae (biobag and loose forms) is carried out by a larger number of specialized laboratories. This competition has led to an increased availability of the method and reduced treatment costs as companies compete for potential clients. The growing demand for the associated assortment translates into a decrease in the overall cost of treatment with larvae, i.e., the profitability of MDT compared to other methods, which has also been observed in Poland in the last decade. For example, the purchase of 100 larvae in a free colony is enough for the debridement of 10–50 cm^2^ of the wound, with an average use of 5–10 larvae per cm^2^, costing 120–200 PLN (30–50 EUR), depending on the direct manufacturer (Biolab^®^ Kędzierzy Koźle or Biomantis^®^ Kraków, Poland) [43]. Larvae in a biobag are more expensive (approx. EUR 80–100). This cost should be multiplied by the area and the number of assumed therapy cycles (usually 2–3). This calculation does not include expenses for the salaries of medical personnel, dressing materials, and visits of the specialist in charge of the treatment. In the study by Soares et al., the cost of MDT treatment was GDP 172.76 per month, and GDP 164.70 in patients treated conventionally with hydrogels [44]. Wayman et al. estimated the cost of medical care, dressings, and larvae at GDP 491.87. For comparison, in this analysis, in the group of patients treated with hydrogels, the total expenses amounted to GDP 1039.53 [45].

### 2.5. Potential Problems Related to Larval Therapy Based on the Analyzed Literature

The general recommendations for the use of MDT/LWT include infected and necrotic wounds of various etiologies in groups of patients in whom typical treatment with surgical necrectomy is not possible, indicated, or for which a moderate benefit is expected. These changes include pressure ulcers with penetrating necrosis, vascular ulcers, and diabetic foot syndrome [44]. Individual case studies indicate the increasing use of biotherapy in infected wounds after surgical interventions in the area of the chest [46], abdomen, and cancer progression, as well as electrical burns [47,48]. The use of maggots as a local therapeutic method should be preceded by the patient’s informed written consent. Larval therapy may have side effects. Bleeding, fever, features of infection, or allergic reaction are potential symptoms that may cause serious systemic disorders. Most experts involved in the implementation of larval therapy into practice point out that it is a safe method, however, in exceptional situations, it may pose a risk of complications (Table 3) [8,9,12,13,15,21].

**Table 3:** Diagnosed health problems and actions taken related to the treatment of a physical wound.

Health Problem	Nursing Interventions	Justification Based on EBM
Increased wound exudate	Explanation of MDT’s action based on the secretion and excretion of digestive enzymes by the larvae, which liquefy the necrosis, resulting in characteristic brown exudate. Inspect the dressing at least every 24 h or more frequently if the exudate is high. Dress the wound with superabsorbent dressings. Dressing inspection should be performed by medical personnel or under their remote supervision, using systems to stay in contact with the person conducting the therapy.	Wound exudate is a physiological phenomenon that occurs at various stages of wound healing. Excess exudate is not desirable and can pose challenges when caring for a patient with a difficult-to-heal wound. Increased exudate and its composition may indicate an infection or contamination/bacterial infection [25,26]. The excessive exudate during MDT therapy is a result of the mechanism of action of maggots in the wound. *Lucilia sericata* larvae produce numerous secretions and excretions that exhibit antibacterial, antibiofilm, anti-inflammatory, and synergistic effects with selected antibiotics [21,26].
Risk of wound bleeding	Selection of the larval application method should be based on the wound’s location, size, and etiology. For wounds located in the abdominal or head areas, it is advisable to use a closed dressing, commonly referred to as a ‘‘biobag’’ or ‘‘biosachet.’’ This precaution is necessary to minimize the potential risk of damaging major blood vessels. In cases where patients have known coagulation disorders or are taking anticoagulants, MDT therapy should be administered with careful consideration, taking into account the patient’s individual advantages and disadvantages. Depending on the patient’s clinical condition, inpatient therapy should be considered, and maintaining open communication with the treating physician is crucial. For outpatient care, it is recommended to change the dressing at least every 24 h, or more frequently if the dressing shows signs of bleeding. The dressing should be regularly inspected by medical staff, with an evaluation of the skin’s condition, the vitality of the larvae, and the patient’s psychophysical state. In cases of active bleeding, apply pressure without restricting the mobility of the larvae. It is important to adhere to the protocols outlined by the Polish Society for the Treatment of Wounds (PTLR) [6,12].	For many years, it was believed that MDT could only be administered to distal body parts. However, the current literature indicates that MDT can also be used in large blood vessels. Patients should be well-prepared for therapy and closely monitored during the process [31]. Patients with deep, penetrating wounds who are on anticoagulants (NOACs or VKAs) are at the greatest risk of bleeding. Antithrombotic treatment does not absolutely contraindicate MDT, but it requires extreme caution [34].
Itching and pain sensations within the wound	Qualification of the patient for therapy after a subjective and objective examination should involve assessing the patient’s suitability for treatment. Patients with a wound-related pain rating exceeding 4 points on the NRS scale and a history suggesting peripheral ischemia require special attention in pain management, particularly concerning hyperalgesia and allodynia. Pharmacotherapy decisions should be tailored to individual patient needs. In cases of hyperalgesia, consideration should be given to either reducing the duration of larvae presence in the wound to 24–48 h or decreasing the number of larvae applied, thus spreading wound debridement over multiple MDT sessions. It is crucial to establish contact with the healthcare provider responsible for the therapy to determine the evacuation of larvae from the wound. Furthermore, future research should focus on the treatment of peripheral arterial disease in patients with an ABPI of < 0.5, a condition often associated with pain, poor vascularization, and concurrent infection.	Contradictory opinions have been presented regarding areas of pain and the perceived effectiveness of therapy. Pain associated with larval activity in the wound can be safely and effectively controlled with the use of pharmacotherapy. In cases of severe symptoms, larvae should be removed from the wound [27,29,30]. In a retrospective study conducted by Mumcuoglu et al. involving 435 patients treated for a total of 723 wounds, it was observed that 38% of patients reported the onset or exacerbation of wound pain during the application of biosurgical dressings. In most cases, pain was managed with analgesics, and only in five cases the issues necessitated a discontinuation of therapy and removal of the dressing [35]. Individuals who are particularly sensitive, have low acceptance of MDT, or have ischemic wounds are more prone to increased pain and require customized preparation for MDT therapy [21,34]. Mudge et al. suggest that factors such as pain management, patient education, and treatment compliance are crucial for enhancing the effectiveness of maggot therapy [34].
Risk of skin irritation around the wound	Increasing the patient’s knowledge about MDT therapy. Clarification of the maggot pattern in the wound, thus possibly increasing wound exudate. Before applying the larvae, the edges of the wound should be protected with a protective paste. Each time the dressing is inspected, the skin around the wound should be assessed, applying additional protective paste as needed. Skin assessment after evacuation of the larvae; in the event of damage, follow the TIMERS scheme when supplying. Proceeding in accordance with the algorithms proposed by PTLR [6].	Excessive exudate related to the mechanism of action of *Lucilia sericata* larvae may determine the maceration of the skin around the wound. The presence of bacteria and proteolytic enzymes may cause micro-damage, which, in the course of contamination, may enlarge the wound area [21,30]. Protection of the wound edges is a key element of activities resulting from the concept of wound hygiene [30,35].

**Figure 3:**
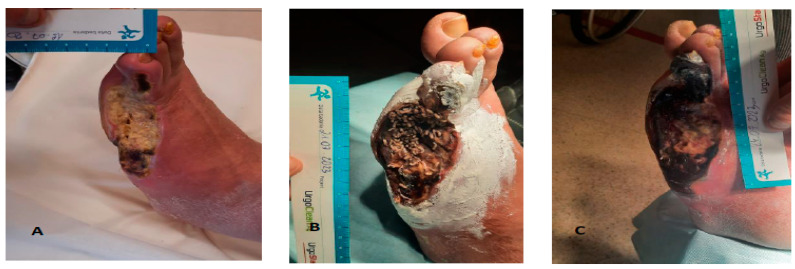
Male, 64 years old, with a history of diabetic ischemic neuropathic foot, after limb revascularization: (**A**): Wound (WIFi 2) before the application of 50 loose larvae, with no bacterial growth in the microbiological assessment, CRP 5 mg/dL, (**B**): the 2nd day of MDT without complications, and (**C**): the 3rd day after the removal of the larvae, showing a fever of 39 °C, signs of tissue undermining, and fulminant necrosis, with CRP at 203 mg/dL.

Despite the simplicity of the method, the authors point out that it is very demanding in terms of supervision over the course of therapy, especially when loose larvae are used in deep and penetrating wounds [21,37,40]. Side effects should be distinguished from complications. Observations and conclusions should be documented. The most frequently described undesirable effects of therapy with the use of larvae are unpleasant sensations such as itching, paresthesia, pain, and bleeding (usually capillary) of varying intensity. Most patients do not feel the presence of larvae in any way (usually in diabetic neuropathy), some report a subjective sensation of wriggling in the wound that does not cause discomfort, while some patients report pain of varying intensity (e.g., patients with ischemic limbs).The risk of hemorrhage increases dramatically, especially in the treatment of the head, neck, limbs, exophytic lesions, or neoplastic ulcers [49]. The occurrences of fever and infection during MDT are sporadic, however, they should be diagnosed as early as possible and qualified after a prior assessment of the patient as a complication of therapy [50,51]. When analyzing the literature, no studies were found that addressed this topic. Jafari et al., in the process of wound debridement in a sample of 80 patients (MDT vs. traditional debridement), showed no difference between the two groups in terms of infection frequency. Inflammatory markers were significantly lowered in the maggot-treated group, indicating less inflammation [52]. The problems associated with the onset of fever and systemic infection are rational concerns for clinicians (Figure 3).

Only selected species of flies (*Lucilia sericata*) can be used in local wound therapy. In order for them to be admitted to medical circulation, their sensation must be limited to dead tissues. More importantly, the eggs laid by the fly should be properly (three times) disinfected with chloramine and sterilized [18,21]. The larva is a living organism well protected by nature against the actions of various bacteria and toxins formed in dead, decaying tissue. Greenberg assumed that antimicrobial compounds could be produced in the maggot gut by symbiotic microbes such as Proteus mirabilis, and in 1986, Erdmann and Khalil recognized two antibacterial substances (phenylacetic acid and phenylacetaldehyde) in Proteus mirabilis isolated from the intestine of a related fly larva: *Cochliomyia hominivorax* [8]. The presence of bacteria in the digestive tract of the larva may lead to wound contamination [53], increasing the risk of infection, especially in ischemic wounds, despite the secretion of large amounts of bactericidal defensins. Out of 238 analyzed questionnaires from patients undergoing MDT at the Podkarpackie Oncology Center (Poland), 6 such cases were found in our unpublished studies.

Patients may be allergic to fly larvae, their media, or the accompanying dressing components. Patients allergic to the maggots or dressing materials may manifest contact dermatitis or more serious immunologic reactions. Larval therapy is not recommended for people allergic to the products used for breeding larvae (brewer’s yeast, soy protein, and hen’s egg white) and for the process of their decontamination. People who are allergic to insect chitin will also not be able to use larval therapy [8,12].

## 3. Discussion

In the analysis of the collected material, a qualitative assessment of the selected literature was conducted in order to identify the potential health problems related to MDT therapy. As a result, most reports indicated that biodebridement is a safe and highly effective method [21,25,26,35]. A small number of studies indicated potential problems mainly related to itching sensations, pain, and disgust at the thought of worms wriggling in the wound [25,28,31,32]. The above observations indicate that the experience and perception of the method in terms of nursing should be defined as a nursing diagnosis.

Since 2012, the Polish standards of education in the field of nursing have indicated that a graduate of BSc studies should know and use the classification of nursing diagnoses. The leading classifications are the International Classification for Nursing Practice (ICNP) and NANDA International (North American Nursing Diagnosis Association). It is worth emphasizing that ICNP belongs to the family of medical classifications of the World Health Organization (WHO) and is recommended by the International Council of Nurses (ICN) and the European Federation of Nurses Associations (EFN) [54,55]. The most important purpose of these classifications is to systematize the current knowledge, universalizing and unifying nursing nomenclature in the country and around the world. When analyzing the subject of the literature, no nursing diagnoses were found that would result from the problems of local therapy with medical larvae.

Despite the established effectiveness of MDT therapy, a considerable number of medical professionals are reluctant to implement this method, considering it controversial. The authors say that fears are mainly due to ignorance and lack of experience [10,15,32]. Educating and raising the awareness of medical personnel are decisive factors that influence the positive perception of this method in the treatment of chronic wounds [14,31,56,57,58]. The factors that increase the level of patient acceptance for therapy with *Lucilla sericata* larvae are mainly the long duration of the difficult-to-heal wound and its destructive impact on quality of life. The described reasons for refusals on the part of patients were female gender, age over 70, and the appearance of maggots [27,29,35,40]. Recent reports have clearly indicated that, during the pandemic period, the necessity for conducting specific procedures in home care has heightened interest in the method of using *Lucilla sericata* larvae for debriding chronic wounds, thus addressing health issues [59]. In the care of a patient, challenges can be categorized into those stemming from psychological sensations and those arising from the physical activities of the larvae within the wound. Negative perceptions and unpleasant mental experiences may intensify physical sensations during therapy [32]. The initial and instinctive response of the patient often involves fear and/or fear resulting from the lack of comprehensive understanding of MDT. The task of the medical staff is to enhance the patient’s understanding of the method, elucidating the entire process while considering both the positive and negative aspects of the therapy.

The authors report that the vast majority of patients want to actively participate in their treatment process. Proper education of the patient will increase their sense of autonomy. The patient–nurse relationship is one of the primary factors influencing patients’ acceptance of therapy. The level of readiness and acceptance of the patient for therapy can be assessed using questionnaire methods [60]. Recognizing health problems enables the planning of medical staff activities, minimizing the negative psychosomatic impacts on the patient. Stressing pro-health behaviors and building the patient’s trust in the person in charge have a positive effect on the willingness to implement innovative local therapy [57,60,61]. Campbell et al. estimated that approximately 25% of patients who were offered biological therapy rejected it [62]. In turn, McCaughan et al., in a qualitative study among patients with wounds, reported that the level of acceptance of MDT therapy was 83%. This resulted from a high desire to heal. Factors influencing positive attitudes included reliable knowledge of the method, care of trained personnel in this field, and family support [27].

Patients commonly feel disgusted by the presence of living creatures in wounds, which are vulnerable due to the broken skin. They associate larvae with poor hygiene, dirt, and perishable food. A reliable explanation of the mechanism of action of maggots in the wound helps to reduce negative thinking in the patient [63]. Campbell et al. reported that the natural stress reactions in patients subsided after the start of therapy [62]. Several authors have suggested that patients’ initial reluctance diminishes during therapy, with patients not experiencing severe symptoms. As they begin to see the positive aspects of the therapy, they willingly recommend this approach to others [8,30,63,64,65,66].

One of the most common negative effects of therapy is pain. Pain perception is an individual matter, resulting from the etiology of the wound and the general condition of the patient. The occurrence of pain or concerns about its severity may negatively influence treatment. Pain as an unpleasant subjective experience is defined as one of the cardinal symptoms, and it may have a complex component (damaged tissues, neuralgia, mental experiences, and blood supply disorders). The identification of patients and implementation of activities related to the assessment of Acceptance (in Poland, the MDT acceptance questionnaire [43,57]) and the assessment of Pain Severity according to NRS/VAS provide the basis for qualifying and preparing patients mentally and physically for the therapy. The intensification of pain was noted in patients who reported pain associated with the wound before MDT therapy. Zarchi and Jemec compared a group of patients whose wounds were debrided with the MDT method and patients whose wounds were debrided using active dressings. The conclusions from the study indicated that the pain occurring in patients who were treated with maggots did not reduce the quality of life of these patients [67]. Diabetic patients present with hypoalgesic sensory disturbances and may not feel pain or are comparable before and after therapy [65,67,68], while patients with ischemic ulcers may poorly tolerate therapy due to hyperalgesia and often require pharmacological preparation before MDT [6,8,60,63,68,69,70]. Mumcuoglu et al., in a cohort study showed, that 38% of patients experienced an increase in pain during therapy, but this pain could be reduced or eliminated by appropriate analgesic treatment before and during the therapy. There is no doubt that a patient with persistent pain must be provided with an analgesic. A holistic perspective should take into account the preferences and acceptance of the method, patients who perceive the sight of larvae negatively should have therapy with closed sets (biobags), and 3D visualizations can also be implemented during dressing changes to distract attention. Appropriately selected pharmacotherapy allows for minimizing negative experiences related to treatment [35]. The sensation of pruritus can occur during therapy, which is also pharmacologically controllable through the use of drugs and subsides when the larvae are evacuated from the wound [35,68]. During MDT, a rarely observed therapeutic effect is the potential for wound bleeding. This effect is connected to the action of proteolytic enzymes on the highly vascularized granulation tissue. Few contraindications of therapy with the use of *Lucilia sericata* larvae include wounds located in the head and abdomen, due to the possible destruction of blood vessels resulting in massive hemorrhage and patients with coagulation disorders taking anticoagulants. However, it should be emphasized that this is not an absolute contraindication, and each patient is individually qualified for biological therapy. The disqualification criterion for MDT therapy is an allergy to chitin, low acceptance of the method, and allodynia [6,57,70].

Inadequate protection of wound edges can result in larvae escaping and the irritation of the skin around the wound [29,30]. An increased amount of exudate from the wound is an additional factor that damages the skin layer. McNichol et al. showed the essence of the MASD phenomenon, i.e., moisture-induced skin damage. The production of exudate is a normal inflammatory response in wound healing, but in excessive amounts, it may contribute to maceration, as well as breaking the continuity of the skin, which is the margin of healthy tissue around the defect [71]. The presence of bacteria, as well as proteolytic enzymes and the non-physiologically increased volume of exudative fluid from the wound bed, significantly reduce the proper barrier functions of the skin, resulting in its soaking. It has been shown that, compared to the exudate from acute wounds, exudate from chronic wounds contains a much higher concentration of proteolytic enzymes that damage the skin layer [72,73]. The extremely often overlooked aspect of the destructive impact of excessive, long-term moisture on healthy tissue should be considered each time MDT therapy is used, in order to minimize the risk of failure and prolong the treatment. To prevent irritation and damage to the skin, the margin of healthy tissue should be protected by simple methods using zinc/stoma paste and external dressings should be changed frequently. However, when irritation occurs, the lesion should be treated in accordance with the guidelines and recommendations resulting from the concept of wound hygiene, in order to avoid widening the lesion area [4,6].

Armstrong et al. highlighted the significant potential of home-based MDT therapy controlled by ICT systems. Clearly pointing to the positive aspects of therapy, they suggest a radically reduced number of appointments and a reduction in the time spent by qualified personnel, contributing to the improvement of the patient’s quality of life. The authors emphasize the safety of home therapy under the supervision of medical personnel [59]. High financial outlays related to the treatment of chronic wounds should lead to the maximum reduction in surgical procedures in hospital conditions. Providing patient care at home with MDT is safe, minimizing the risk of infection and implementing antibiotic therapy. A condition that will significantly affect the quality of biological therapy in a patient with chronic wounds will be the standardization of the practice of using MDT at home.

## 4. Conclusions

Local therapy using *Lucilia sericata* larvae for wound debridement and tissue revitalization holds promise as a prospective method for the future, despite potential health concerns related to its specific nature. Its ability to rapidly debride wounds and reduce microbial contamination makes it a viable recommendation. However, concerns related to visual and olfactory experiences, as well as pain sensations, may influence the perceptions of both patients and medical personnel, potentially impacting the adoption of this method. Educating healthcare professionals about the benefits for patients is crucial for changing their attitude towards implementing innovative local wound treatment methods. Further research should be conducted to report adverse events and complications, integrate health economic evaluations, and conduct parallel qualitative studies to better understand the cost-effectiveness, acceptability, and patient experiences of larval therapy.

### Limitation

Our analysis was based on the available literature concerning health problems in patients with chronic wounds undergoing larval therapy. The limited number of scientific papers available prevented a comprehensive analysis of the discussed issues.

## Figures and Tables

**Figure 2:**
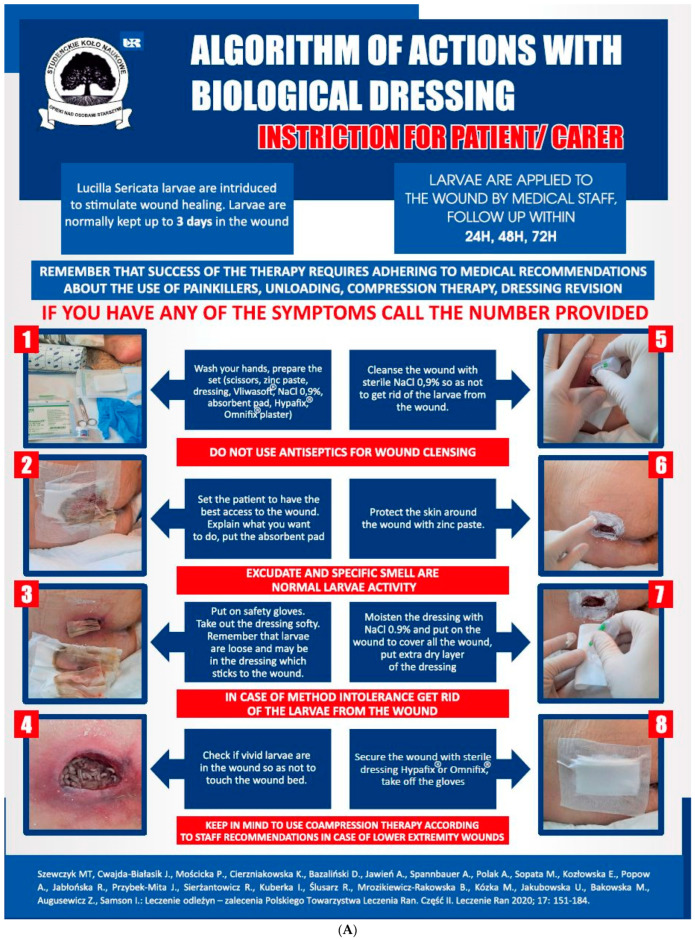

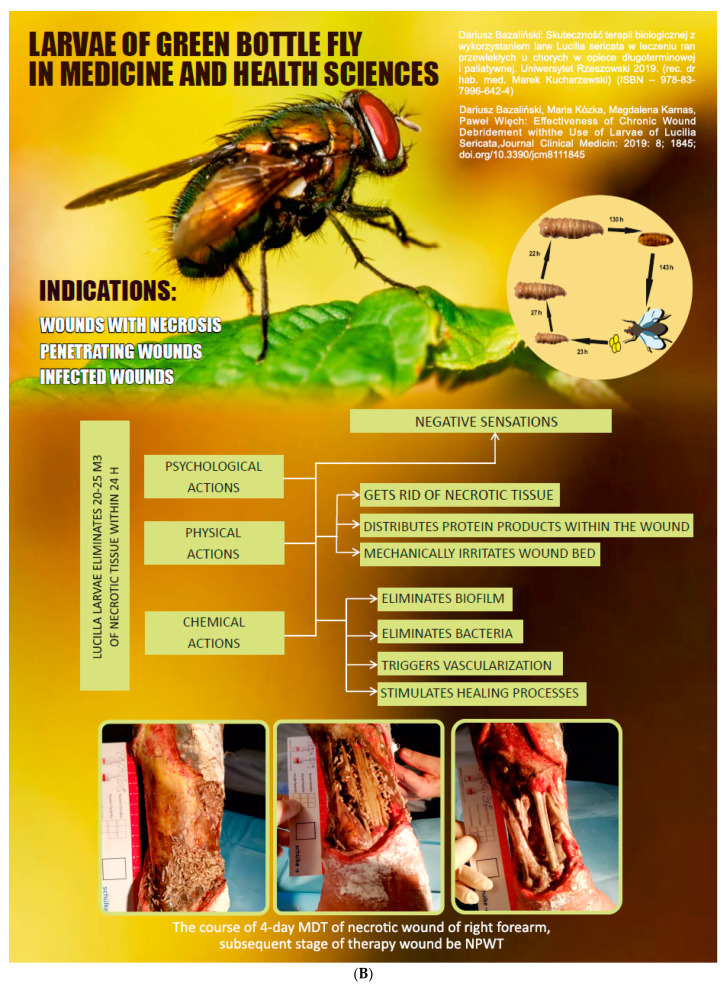
Posters prepared for educational purposes in the wound treatment clinic at the Podkarpackie Oncology Centre: (**A**) dressing application and supervision process, and (**B**) action of larval therapy in the wound [8].

**Table 1:** List of analyzed works.

Author	Type of Work	Participants	Conclusion
Opletova et al. (2012) [21]	Randomized controlled trial	105 patients with lower leg ulcers ABPI ≥ 0.8, divided into two groups.	MDT showed no significant benefit on day 15 compared to conventional treatment. Debridement with MDT was much faster and took place in the first week of treatment. Another type of dressing should be used after 2 or 3 applications of MDT. Pain scores were similar and low in both groups.
Parajilio et al. (2021) [25]	Original article	Qualitative study via information provided by the Biotherapeutics, Education, and Research Foundation (BTER) website. Structured telephone interviews were conducted with nine healthcare professionals who use MDT.	Specifically, the ‘yuck’ factor and the perception of MDT as an ‘ancient’ modality contributed to MDT stigma; in addition, a lack of outpatient insurance coverage deterred MDT use.
Dumville et al. (2009) [26]	Randomized controlled trial	267 patients with mixed lower leg ulcers ABPI ≥ 0.6, divided into two groups.	Larval therapy did not improve the healing time of the lower leg ulcers or reduce the number of bacteria compared to the hydrogel, but it significantly shortened the time required for wound cleansing. An increase in pain sensations during MDT was noted.
McCaughan et al. (2015) [27]	Randomized controlled trial	18 people (12 men, 6 women) aged from 29 to 93 years (median age 64) with at least one venous leg ulcer. Most were willing to try the “worms” (larvae) and were able to overcome the feeling of disgust because they wanted to heal their wounds.	Patients may hold unrealistic expectations that larval therapy will effect a longed-for cure for their leg ulcer(s), but an absence of healing may lead to feelings of disappointment or despair.
Morozow et al. 2019 [28]	Original article	A total of 576 people were enrolled: 414 (72%) women and 162 (28%) men. Patients’ perception of the sight of maggots and larval therapy were assessed.	Maggots are repulsive to many people, and this may influence the acceptance of larval therapy.
Spilsbury et al. (2008) [29]	Original article	The preferences of 35 patients regarding the acceptance of two forms of larval therapy (“in biobags” and “loose”) were assessed.	Eliciting patient preferences and increasing their involvement in treatment decisions are important elements for improving quality and achieving better health outcomes. These findings have implications for practitioners offering larval therapy as a treatment option and for the feasibility of clinical trials.
Turkmen et al. (2010) [30]	Original article	Larval therapy was implemented in 34 patients with chronic wounds; in 29 (85%), satisfactory wound cleansing was observed. In the remaining five patients, failures occurred due to insufficient dressing sealing in two patients (6%), death of larvae in two patients (6%), and method intolerance in one patient (3%).	Larval therapy should be considered as a therapeutic option for the management of certain challenging wounds.
Przybek-Mita et al. (2022) [31]	Original article	The study group consisted of 290 nurses specialized in chronic wounds undergoing training out of an entire group of 1136 individuals participating in training courses organized in Poland in 2020–2021.	The level of perceived stress may influence decisions related to the use of biological therapy. The higher the level of stress, the lower the readiness to undertake MDT.
Hopkins et al. (2022) [32]	Original article	The first stage of this mixed-methods study was a focus group organized to discuss MDT and the opinions of specialist nurses. An anonymous online survey was then launched via Nursing Times and distributed on social media to all nurses. Finally, in-depth interviews were conducted with specialist and general nurses.	Nurses specializing in wound care choose MDT more often than nurses without experience in this field. Attention was paid to the need for nurse training to solve problems with acceptance and willingness to use this method in practice.
Nigam et al. (2022) [33]	Original article	In a group of 412 respondents regarding the acceptance of larval therapy, only 36% of the respondents agreed to accept larval therapy as a first-line treatment in the case of a hypothetical painful wound.	Study participants expressed concerns and fears related to the use of larval therapy. Positive associations were reported between knowledge scores and potential acceptance of larvae therapy, suggesting that information dissemination and education may be an important factor influencing public perception and acceptance of the method.
Mudge et al. (2014) [34]	Randomized controlled trial	88 patients with venous and mixed ulcers ABPI ≥ 0.5 divided into two groups.	Larval therapy cleansed ulcerative wounds faster than hydrogel. Patients in the larvae group experienced more discomfort and pain during wound cleansing compared to the hydrogel group.
Mumcuoglu et al. (2012) [35]	Original article	Of the 435 patients who underwent MTD, 165 (38%) reported increased pain. In five patients, treatment had to be discontinued due to uncontrolled pain.	A large percentage of patients treated with MDT reported increasing pain with each day of therapy. It is necessary to introduce painkillers when cleaning with larvae.

**Table 2:** Diagnosed health problems and actions taken related to the treatment of a wound.

Health Problem	Nursing Interventions	Justification Based on EBM
Repulsion and disgust caused by the sight of larvae wriggling in the wound	Increase patient knowledge about Medical Maggot Therapy (MDT) to reduce negative feelings. Reinforce positive health behaviors and focus education on the positive effects of rapid wound debridement with minimal discomfort. In order to gauge patient acceptance, it is recommended to use a questionnaire assessment, such as the Perceived Stress Scale (PSS10) to assess stress levels and an MDT acceptance assessment. Explain the mechanism of action of the larvae in the wound and the potential use of a “biobag” to reduce visual sensations. Implement procedures to limit the view of maggots in the wound, such as using posters (see Figure 2), brochures, and instructional videos. Facilitate direct communication with the healthcare provider who administers the therapy to reduce patient anxiety. After completing the therapy, encourage patients to assess the effects of wound debridement.	Patients who consented to the MDT application re-evaluated their initial expectations compared to the post-treatment period. Positive opinions about the therapy persisted; small larvae in the first growth phase did not frighten the patients [25,26,27]. MDT experiences and interpersonal relationships influenced the perception and interactions between the patients and other individuals, including medical staff, friends, family, and fellow patients. The longer the problems with wound healing lasted, the higher the acceptance of larval therapy [28].
Anxiety about the larvae leaving the wound	Explanation of application methods, including both closed and open techniques, and highlighting the potential benefits of each method, can help to address anxiety about the larvae leaving the wound. Ensuring continuous contact with a dedicated healthcare professional responsible for the treatment is essential. To further mitigate concerns, sealing the wound with an appropriate dressing and having medical staff regularly inspect the dressing for patients who may not be self-reliant, or implementing remote supervision using audio-visual techniques, are all recommended strategies.	Anxiety related to the fear of larvae leaving the dressing was discussed by Turkmen [30] and Sherman and Morozov [28]. Nursing staff’s uncertainty contributes to patients’ apprehension regarding the adoption of an unfamiliar therapy. Building confidence in their actions instills a sense of security in patients, reducing their anxieties both before and during MDT therapy [28]. Encouraging patient preferences and enhancing their involvement in treatment decisions are crucial components of quality improvement and better health outcomes. These findings hold significance for practitioners offering larval therapy [25,27,28]. Medical staff may also experience anxiety, especially concerning the appearance and movement of larvae, potentially leading to their hesitancy in using this method [31].
Lack of motivation to engage in health-related actions and resistance to treatment	If possible, assess the need for support, motivation, and self-care capabilities. In cases of the absence or limited functional resources, consider long-term care options and enhance collaboration with primary healthcare providers and caregivers.	Older age, loneliness, and limited self-care can lead to a decrease in or lack of motivation for health-related activities [32]. People subjected to larval therapy whose wounds did not heal for a long time showed a positive attitude and trust in wound healing [29]. In Nigam et al.’s study, 36% of the participants agreed that they would accept larval therapy as a first-line treatment for a hypothetical painful wound, although this number increased with wound severity. The most dominant concerns regarding larval therapy were the sensations and feelings of disgust associated with the therapy [33].

## Data Availability

The data presented in this study are available on reasonable request from the corresponding author: p.k.wiech@gmail.com.
